# Marriages and Deaths in Buffalo

**Published:** 1848-11

**Authors:** 


*Marriages and Deaths in Buffalo.*—The Buffalo Commercial Advertiser gives the following statement of marriages and deaths in this city for the year ending August 8th, 1848. The data, it is stated, have been obtained by Mr. E. W. Palmer, from Clergymen, Justices of the Peace, Sextons, &c.

The whole number of marriages is 791. Of this number, 501 have been by the German and French clergymen of the city.

The whole number of deaths, as reported by the several sextons, is 1,499. In addition to this number, it is estimated that there were about 50 private burials, This makes an average of about 30 per week; or, one in 1500, estimating our population at 45,000.

The Advertiser adds, that "during the time embraced, the mortality of the city has been made greater than usual by temporary causes. The ship fever aud small pox prevailed to considerable extent last autumn, and carried off quite a large number soon after their arrival here, who had not become citizens. This is shown in the report of between 300 and 400 paupers, who were buried at the expense of the city."

A complete, systematic registration of births, marriages, and deaths in this city is highly desirable. If not provided for by the present law on the subject, the act should be properly amended, and it should not be permitted to become a dead letter.

